# Pedestrian Crossing Sensing Based on Hough Space Analysis to Support Visually Impaired Pedestrians

**DOI:** 10.3390/s23135928

**Published:** 2023-06-26

**Authors:** Takeru Yoshikawa, Chinthaka Premachandra

**Affiliations:** 1 Electrical Engineering and Computer Science, Graduate School of Engineering and Science, Shibaura Institute of Technology, Tokyo 135-8548, Japan; chintaka@sic.shibaura-it.ac.jp; 2 Department of Electronic Engineering, School of Engineering/Graduate School of Engineering and Science, Shibaura Institute of Technology, Tokyo 135-8548, Japan

**Keywords:** visually impaired people, pedestrian crossing sensing, walking support system, Hough space analysis, parallel line extraction

## Abstract

There are many visually impaired people globally, and it is important to support their ability to walk independently. Acoustic signals and escort zones have been installed on pedestrian crossings for the visually impaired people to walk safely; however, pedestrian accidents, including those involving the visually impaired, continue to occur. Therefore, to realize safe walking for the visually impaired on pedestrian crossings, we present an automatic sensing method for pedestrian crossings using images from cameras attached to them. Because the white rectangular stripes that mark pedestrian crossings are aligned, we focused on the edges of these rectangular stripes and proposed a novel pedestrian crossing sensing method based on the dispersion of the slope of a straight line in Hough space. Our proposed method possesses unique characteristics that allow it to effectively handle challenging scenarios that traditional methods struggle with. It excels at detecting crosswalks even in low-light conditions during nighttime when illumination levels may vary. Moreover, it can detect crosswalks even when certain areas are partially obscured by objects or obstructions. By minimizing computational costs, our method achieves high real-time performance, ensuring efficient and timely crosswalk detection in real-world environments. Specifically, our proposed method demonstrates an impressive accuracy rate of 98.47%. Additionally, the algorithm can be executed at almost real-time speeds (approximately 10.5 fps) using a Jetson Nano small-type computer, showcasing its suitability as a wearable device.

## 1. Introduction

**Figure 1:**
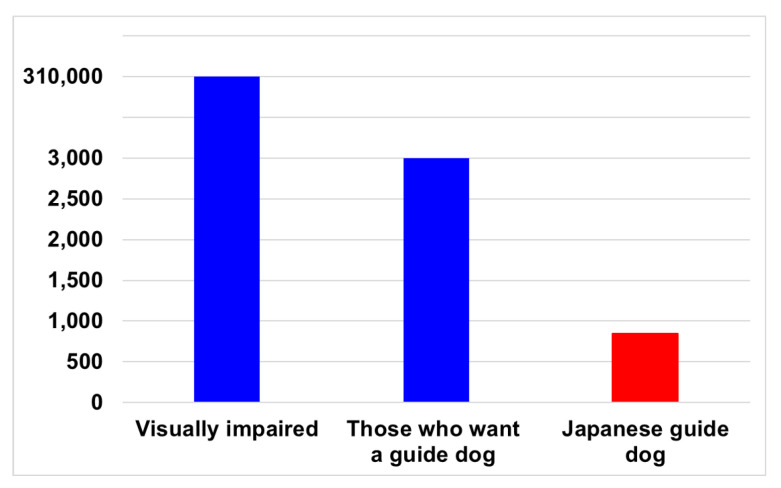
The number of visually impaired people and guide dogs in Japan in 2022.

**Figure 2:**
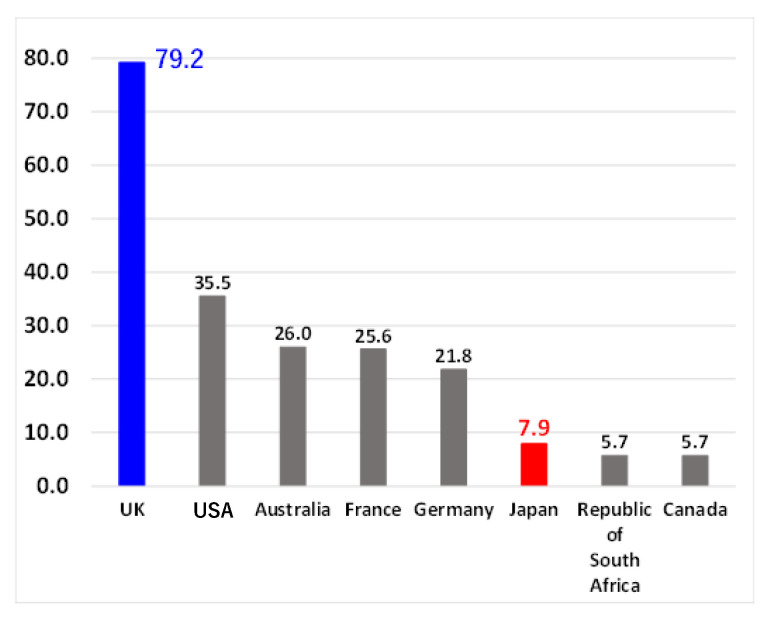
The numbers of guide dog users per million people in different countries in 2013.

There are many visually impaired people in the world, including Japan. For instance, in 2022, the number of visually impaired people in Japan was approximately 310,000, and the number of people who required guide dogs was approximately 3000. This has been illustrated in Figure 1. However, there are only 848 guide dogs currently being used in Japan [1]. Furthermore, as shown in Figure 2, when the number of guide dog users per one million people is compared by country, this number is extremely small [2]. In particular, when compared with the United Kingdom, there is an approximately 10-fold difference. If the utilization rate of the United Kingdom was to be realized in Japan, the number of guide dog users in Japan would be approximately 10,000, which would meet the needs of all guide dog applicants. In other countries, the number of guide dog users is small in relation to the number of visually impaired people, and the environment in which the visually impaired people can move freely is underdeveloped.

The low usage level of guide dogs in Japan can be attributed to the differences in sidewalks, breeding environments, and a lack of social understanding.

Therefore, there is a need for walking supports that do not rely on living organisms. Two types of devices have been used to improve safety at crosswalks, where accidents are particularly common. The first is an acoustic traffic signal with a device that emits a guiding sound. The second is an escort zone with braille blocks on the crosswalk. These devices and considerations to achieve safe crosswalks for all pedestrians, including the visually impaired, have become widespread.

However, accidents involving the visually impaired continue to occur at crosswalks. This can be because the acoustic traffic signals do not emit sound from night to morning owing to noise considerations. Additionally, the escort zone deteriorates, and the unevenness is worn away.

**Figure 3:**
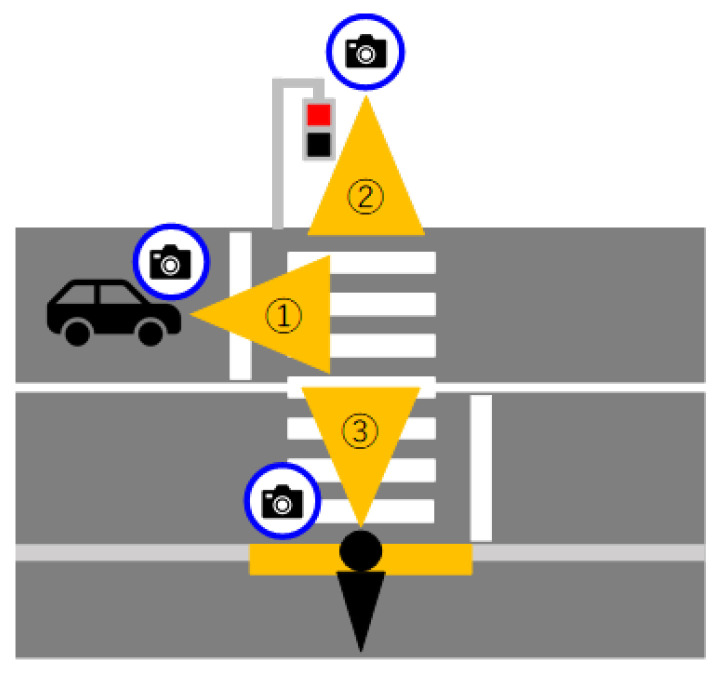
Camera-based pedestrian crossing detection.

Therefore, as shown in Figure 3, there are three methods outlined in this study: pedestrian-mounted cameras [3,4], in-vehicle [5,6,7,8,9,10,11,12], and field fixed [13,14], and pedestrian-mounted cameras. However, in-vehicle cameras and fixed-field crosswalk detection methods are not useful for walking support systems.

We established that it is possible to realize safer walking when a visually impaired person can perform the detection directly using a pedestrian-mounted system. Therefore, we propose a wearable pedestrian system.

In order to overcome the limitations of existing methods, we focused on developing a detection method specifically designed for pedestrians to wear [15,16,17,18,19,20,21,22,23,24,25,26,27]. This innovative approach addresses the challenges associated with conventional methods. One such challenge is the dominance of experiments conducted during the daytime, which often results in crosswalk images lacking pedestrians obstructing the view. This limitation restricts the applicability of the existing methods in real-world scenarios. Additionally, there is a scarcity of methods capable of efficient real-time processing, further impeding their practical usability.

**Figure 4:**
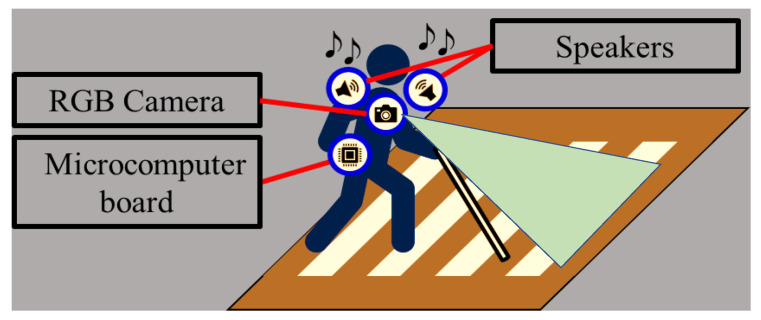
Application of the proposed pedestrian crossing detection method.

To tackle these challenges, our study introduces a cutting-edge pedestrian-mounted (Figure 4) crosswalk detection method. Our primary goal was to not only address the aforementioned limitations but also create a hardware solution that is user-friendly, portable, and lightweight, ensuring ease of use and convenience for pedestrians.

The core principle of our proposed method lies in leveraging the fact that crosswalks typically exhibit a distinctive pattern of continuous white rectangular shapes. By utilizing the Hough transform method [28], we enhanced the contours generated by a Canny edge detector [29] and the shapes of these rectangular patterns, enabling the accurate detection of crosswalks based on the variations in the slope of the straight lines associated with the contours in Hough space.

The unique characteristics of our proposed method enable it to handle various challenging scenarios that traditional methods struggle with. For instance, it can effectively detect crosswalks in low-light conditions during nighttime, where illumination may vary. Furthermore, the method is adept at detecting crosswalks even when certain parts of the crosswalk are partially obscured by objects or obstructions. Importantly, our method achieves an almost real-time performance by minimizing computational costs, ensuring the efficient and timely detection of crosswalks in real-world environments.

The proposed method demonstrates a high accuracy rate of 98.47%. Additionally, the algorithm can be executed almost in real-time (approximately 10.5 fps) using a Jetson Nano small-type computer, which highlights its applicability as a wearable device.

## 2. Related Work

Many studies have been conducted on supporting visually impaired with wearable devices [3,4], including crosswalk detection. Although the goal is to detect crosswalks, the objects to which the cameras are attached vary significantly. There are three main types of cameras: in-vehicle, fixed-in-place, and pedestrian-mounted cameras.

These cameras have different objectives and results. First, for the in-vehicle cameras, there are many studies, including those of Yuhua Fan et al. [5], J. Choi et al. [6], and many more [7,8,9,10,11]. These cameras aim to detect pedestrians on the crosswalk. However, it is difficult to put them into practical use because detection is insufficient. In addition, they were developed considering the perspective of the vehicle and not that of the visually impaired persons.

Second, there are many examples of detecting crosswalks using a fixed camera installed near a crosswalk [12,13]. The goal of these systems is to detect pedestrians on a crosswalk using a surveillance camera located at the site. They are not useful in assisting the visually impaired in walking because they cannot provide guidance.

Therefore, because it is difficult for the in-vehicle and fixed-in-place cameras to provide walking support for the visually impaired, we aim to realize safer and more accurate walking by allowing visually impaired individuals to achieve it directly.

Therefore, a pedestrian wearable system is proposed. There are several studies on pedestrian crossing detection, including the work of Ruiqi Cheng et al. [14] as an example of a similar method. The collection of papers encompasses a wide range of innovative research efforts aimed at improving the detection and recognition of marked pedestrian crossings in various contexts, with a specific emphasis on addressing challenging scenarios and catering to the needs of individuals with visual impairments. Wu et al. [15] propose a block-based Hough transform approach that effectively identifies marked crosswalks in natural scene images, contributing to the development of robust detection methods. Radványi et al. [16] introduce advanced crosswalk detection techniques tailored for the Bionic Eyeglass, offering enhanced functionality and usability for visually impaired users. Cao et al. [17] present an image-based detection method specifically designed for pedestrian crossings, utilizing visual cues and patterns to identify these critical areas. Akbari et al. 18] propose a vision-based marked crosswalk detection method that caters to the unique needs of individuals with visual impairments, empowering them with improved mobility and safety. Mascetti et al. [19] introduce ZebraRecognizer, an advanced pedestrian crossing recognition system explicitly developed for individuals with visual impairment or blindness, offering real-time assistance and guidance. These papers collectively demonstrate a broad spectrum of approaches, including the integration of computer vision applications into wearable devices (Silva et al. [20]), leveraging the ubiquity of camera phones for crosswalk detection (Ivanchenko et al. [21]), employing sophisticated SVM-based column-level approaches for accurate detection in low-resolution images (Romić, K. et al. [22]), and harnessing the power of deep convolutional neural networks for the precise identification and localization of marked crosswalk areas (Haider et al. [23]). Furthermore, the collection includes papers exploring diverse areas, such as image analysis techniques for crosswalks (Shioyama et al. [24]), the development of lightweight semantic segmentation networks for the rapid detection of blind roads and crosswalks (Cao et al. [25]), the creation of crosswalk guidance systems for the blind (Son et al. [26]), and the utilization of RGBD cameras for detecting both stairs and pedestrian crosswalks (Wang et al. [27]). These comprehensive efforts contribute to certain improvements in crosswalk detection technology, but its practical application level is still poor in terms of achieving pedestrian safety, especially for individuals with visual impairments. One major reason for this is that the detection rate is not very high, particularly when a part of the crosswalk is obscured or during nighttime.

Therefore, based on the results of previous studies, this study proposes a method for the automatic detection of crosswalks from camera images worn by the visually impaired to realize safe walking at crosswalks. Furthermore, as shown in Figure 4, the proposed method can be implemented using small and lightweight hardware that can be worn and easily be carried by the user. The proposed method exhibited a higher accuracy than any other method reported in the literature.

## 3. Description of the Pedestrian Crossing Detection Method

### 3.1. Outline of Application

Figure 4 shows the application of the proposed pedestrian-crossing detection method. The method aims to detect the pedestrian crossing using images from the camera worn by the pedestrian and guide them across the crosswalk via audible cues.

Pedestrian crossings are designated as white rectangular stripes. Therefore, in this study, we focused on the edges of white rectangular regions and propose a novel pedestrian crossing detection method based on the variance of the slope of a straight line in Hough space formed by the white stripes.

### 3.2. Image Acquisition

Images can be acquired using various two-dimensional cameras. Table 1 lists the specifications of the camera used in this study. We used the camera in an iPhone 7 to conduct the experiment.

**Figure 5:**
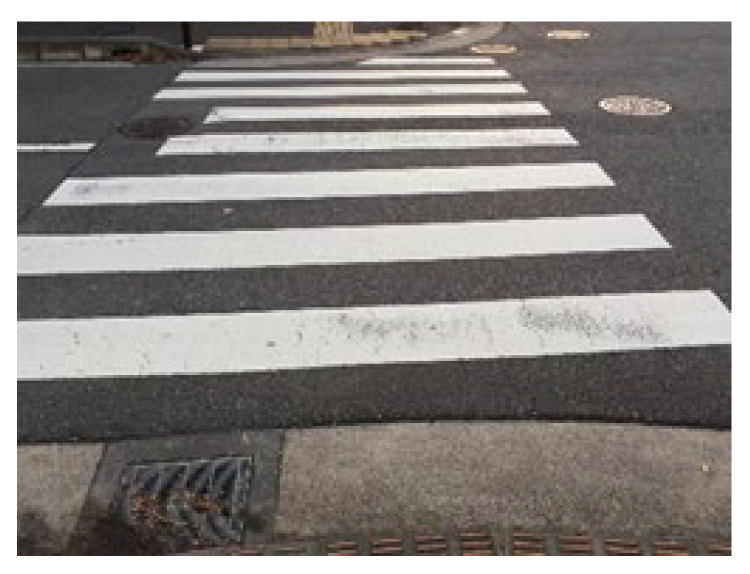
Input image acquired by the camera.

Figure 5 shows the input image obtained by the camera. As shown in Figure 4, the camera was fixed to the chest of the pedestrian and faced forward. The width of the pedestrian crossing was approximately 10 m, and video acquisition was begun approximately 3 m in front of the pedestrian crossing. In addition, because we assumed the need for support while walking, images were captured continuously.

## 4. Pedestrian Crossing Detection

### 4.1. Flow of the Proposed Pedestrian Crossing Detection Method

**Figure 6:**
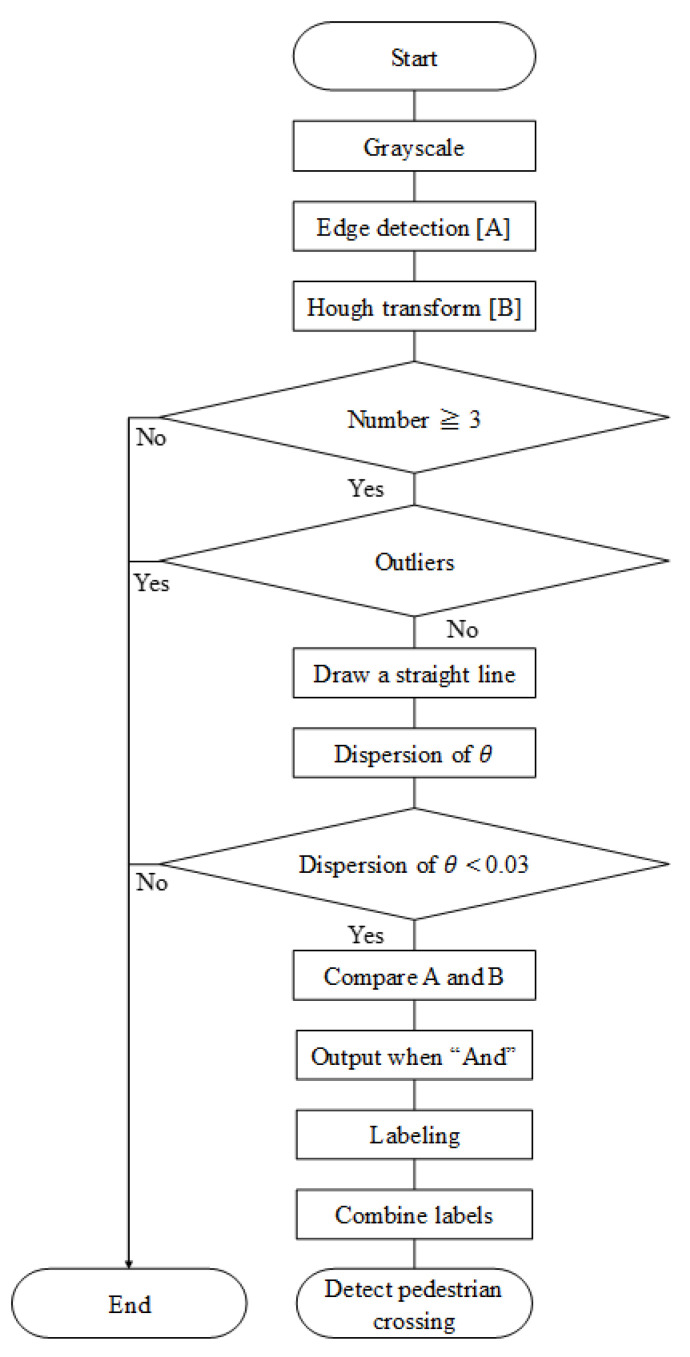
Loop flow of the proposed pedestrian crossing detection method.

The process flow of the proposed automatic crosswalk detection method is shown in Figure 6. Here, we provide a summary of the overall processing flow in the following sentences. First, the acquired image was grayscaled. Second, edge detection was performed using the Canny edge detection method. Thereafter, Hough transform was performed on the image to detect the straight-line part of the edge. When there are more than three straight lines obtained via Hough transform, we continued the process as a candidate for the crosswalk area. Three or more straight lines are selected here because the width of the three straight lines is approximately 1 m, which can be used as a guide to maintain safety. Subsequently, the slope of the straight lines is measured, and only the lines that are close to the slope are drawn. Here, the extraction of the drawn lines is based on the variance in the inclination of the lines. We varied the threshold value and adopted 0.03 because it had the highest accuracy in many experiments. The drawn lines were then compared with the original edge image, and the range of the crosswalk was extracted. Finally, the labeling process was performed, and the detected labels, except for those that were significantly smaller, were combined to determine the crosswalk area, completing the detection of the crosswalk. The above content pertains to the overall process of the proposed method. Detailed information on the major stages shown in Figure 6 is presented in the subsections below.

### 4.2. Edge Detection

This section presents the content regarding edge detection shown in Figure 6, including the grayscaling process. The Canny method, which is often used for preprocessing to recognize objects in an image [30,31,32,33,34], was used to detect edges in the moving image. The Canny method was classified into five stages.

First, smoothing was performed using a Gaussian filter to weigh the pixel values around the pixel to be processed as well as the Gaussian distribution. The relationship between the input image, I; Gaussian filter kernel, $K_{g}$; and smoothed image, G, is given according to Equation (1). * denotes a convolution integral.

(1) $$G=I*K_{g}$$

Second, the smoothed image G was differentiated using the Sobel filter [35]. The relationship between the horizontal differential kernel, $K_{x}$, of the Sobel filter; vertical differential kernel, $K_{y}$; horizontal differential image, $G_{x}$; and vertical differential image, $G_{y}$, is given according to Equations (2) and (3).

(2) $$G_{x}=G*K_{x}$$

(3) $$G_{y}=G*K_{y}$$

Third, the gradient magnitude, $\left|G\right|$, and direction θ were obtained from differential image G according to Equations (4) and (5), respectively.

(4) $$\left|G\right|=\sqrt{G_{x}^{2}+G_{y}^{2}}$$

(5) $$\theta =\tan^{-1}\frac{G_{y}}{G_{x}}$$

Fourth, the contour of the differential image $\left|G\right|$ was thinned via non-maximum suppression processing. Specifically, the pixel value of the pixel of interest is compared with the pixel value adjacent to the gradient direction of the contour; if the pixel value of the pixel of interest is not the maximum, the pixel value is not considered an edge pixel.

Finally, hysteresis threshold processing was used to select reliable contours and unreliable contours based on the maximum and minimum thresholds, and only highly reliable contours were drawn. Specifically, this stage classifies contours into three types. First, a reliable contour is used when the pixel value is larger than the maximum threshold. Second, the contour is considered unreliable when the value is smaller than the minimum threshold value. Finally, if it is between the maximum and minimum thresholds, the contour is reliable if adjacent contours have high reliability and vice versa. In this study, the maximum and minimum thresholds were set to 300 and 50, respectively.

**Figure 7:**
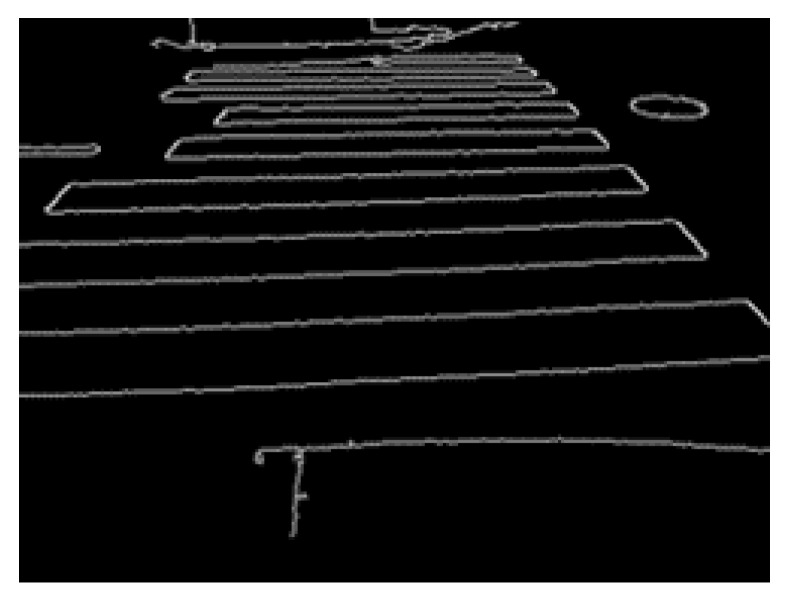
Edge image via the Canny method.

The result of this process is presented in Figure 7.

The Canny method, which performs the above processing, is characterized by less false detection and the non-detection of contours than the Sobel filter and Laplacian filter, which are also used for edge detection.

### 4.3. Drawing Straight Lines by Detecting the Edge Components Related to Them

Following Figure 6, we utilized Hough transform to detect straight lines representing edges. Once the lines were detected, we proceeded to draw solid lines on them using the equations. Hough transform is widely used in object recognition from images. Generally, Equation (6) is used to show the parameters of a straight line.

(6) y=ax+b

However, when the slope of the line is parallel to the *y*-axis, the slope becomes ±∞, and an unnecessary intercept information is used.

**Figure 8:**
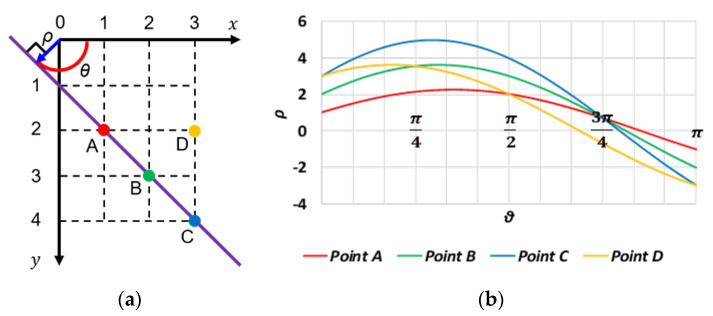
Hough transform example. (**a**) Straight line in Hough space. (**b**) Spatial plot of $\left(\theta ,\;\;\rho \right)$.

This is where Hough transform occurred. As shown in Figure 8, Hough transform is a method that performs calculations in the $\left(\theta ,\;\rho \right)$ space with linear distance ρ from the origin of each pixel and angle θ between that line and the x-axis. In this space, it is represented according to Equation (7). In Figure 8, points A, B, and C lie on a straight line, while point D does not.

(7) ρ=x·cosθ+y·sinθ

According to Equation (7), countless lines pass through a pixel. However, the coordinates $\left(x,\;y\right)$ of a pixel can be fixed to represent a straight line between pixels. In addition, as angle θ changes, the corresponding linear distance ρ from the origin to each pixel is determined using a single solution. Therefore, we only need to consider one dimension. Thereafter, considering the symmetry of the figure, we can find the same $\left(\theta ,\;\rho \right)$ in several pixels if we find the solution of $\left(\theta ,\;\rho \right)$ in the range of 0≤θ≤π. This means that the parameters of the function of the straight line in Hough transform are the same. That is, pixels on the same line have the same $\left(\theta ,\;\rho \right)$.

Therefore, if we calculate $\left(\theta ,\;\rho \right)$ for all edge pixels and plot them with angle θ on the horizontal axis and linear distance ρ from the origin to each pixel on the vertical axis, the curves of the pixels on the same line will have the same $\left(\theta ,\;\rho \right)$ and will thus intersect. The greater the number of curves at the intersection, the more reliable the straight line is.

There are two types of Hough transform: ordinary [36,37,38,39] and probabilistic [40]. The former has a high detection rate of straight lines because it calculates for all pixels, but it requires more processing. In the latter method, the minimum number of pixels required for straight-line detection is selected arbitrarily for calculation, and the straight-line detection rate and the processing time are low.

**Figure 9:**
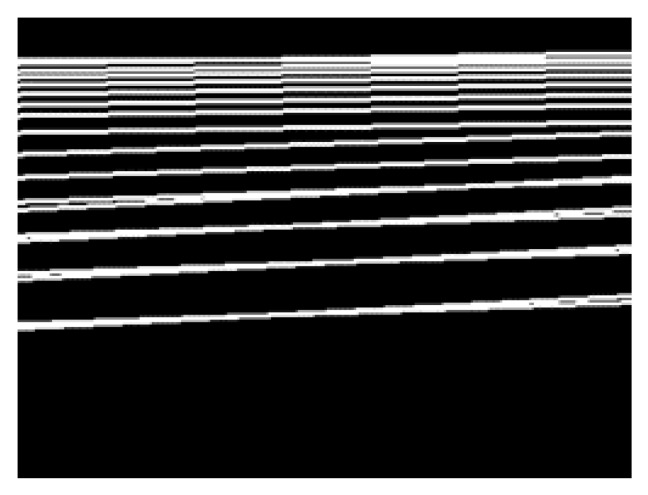
Image of straight line obtained through Hough transform.

In this study, the probabilistic Hough transform of the latter method did not provide a sufficient detection rate; therefore, the ordinary Hough transform of the former method was used. Figure 9 shows an image in which only the straight lines obtained through Hough transform are extracted.

.

### 4.4. Extraction of Parallel Lines Based on Variance in Angle Information in Hough Space

The contents of this section pertain to the calculation of dispersion (variation) for parallel lines, as indicated in Figure 6. The white rectangular stripes in a pedestrian crossing have parallel boundaries. Therefore, if the variance value, $S^{2}$, of the angle is calculated using Equation (8) from the information on the angle of the straight line obtained through Hough transform in the previous section, $S^{2}$ will be zero in the ideal state.

Therefore, when $S^{2}$ is close to zero, the area is recognized as a pedestrian crossing. However, $S^{2}$ does not completely approach zero in reality. In this study, the threshold value, $S_{th}^{2}$, of $S^{2}$ was set to 0.03 from the experimental value because linear distortion exists due to perspective and other noise.

$S^{2}$ was obtained from the information on the angle of the straight line obtained via Hough transform, and when the value satisfied Equation (9), the lines are processed to represent a pedestrian crossing.

(8) $$S^{2}=\frac{1}{n}\overset{n}{\underset{n=1}{\sum }}{\left(\theta_{i}-\bar{\theta }\right)}^{2}$$

(9) $$S^{2}<0.03$$

### 4.5. Combining Edge Image and Hough Transform Image

**Figure 10:**
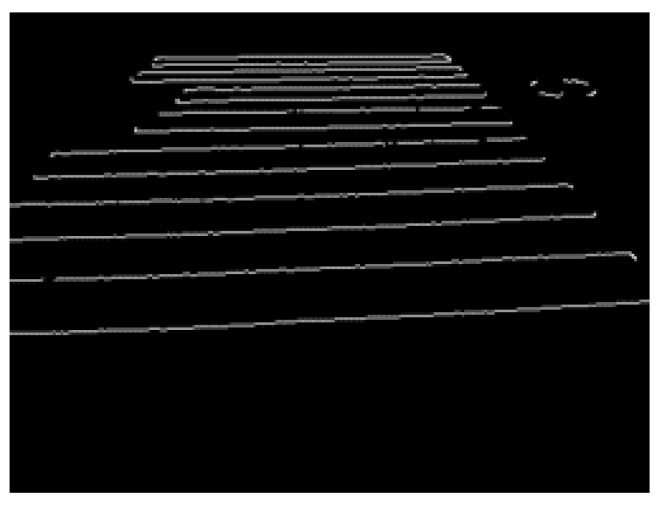
Composite image.

In this section, we primarily focus on the combining process of the edge image and the Hough transform image, following a comparison between the two, as depicted in Figure 6. The edge image (Figure 7) and Hough transform image (Figure 9) were compared to extract only the pedestrian crossing area. Here, the Hough transform image is a line drawn by detecting a straight line from the edge image using Hough transform. Both are binarized images. Therefore, from Equations (10) and (11), only the pixels that hold the logical product of both the edge image $G_{E}$ and the Hough transform image $G_{H}$ are extracted. With this processing, the straight part of the pedestrian crossing in the edge image can be extracted. The results of this processing on the edge image in Figure 7 are presented in Figure 10.

However, if there is an edge other than the pedestrian crossing in the extension of the edge of the pedestrian crossing, the edge will be output even though it is minute. This part was removed during the labeling step.

(10) $$G_{E}\wedge G_{H}=255$$

(11) $$\bar{G_{E}\wedge G_{H}}=0$$

### 4.6. Labeling

To determine the crosswalk area, we performed labeling on the combined image of the edge image and the Hough transform image. Labeling is a process of concatenating consecutive output pixels in a binarized image and assigning the same number to them.

**Figure 11:**
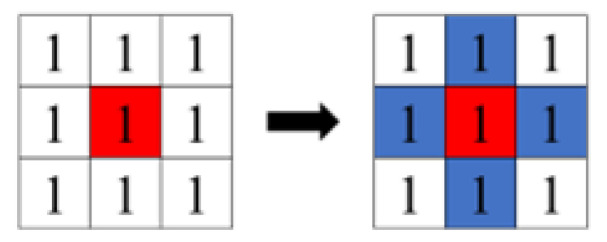
Four concatenations.

**Figure 12:**
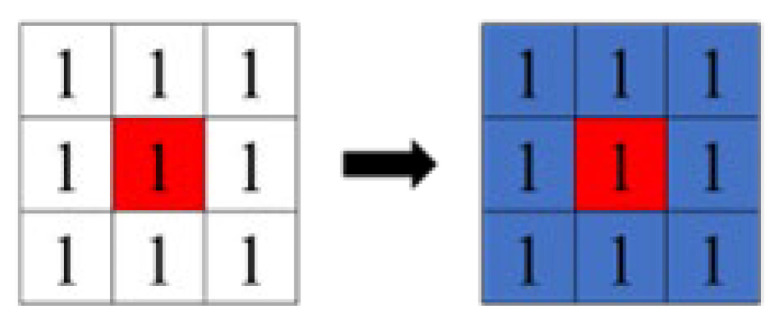
Eight concatenations.

There are two types of labeling: four-connected (Figure 11), which gives the same label to consecutive pixels in the vertical and horizontal directions of the binarized image, and eight-connected (Figure 12), which gives the same label to pixels connected in the vertical, horizontal, and diagonal directions. The red area in the figure represents the pixels of interest. In this study, we used the eight-connected method, which allows labeling in the diagonal direction in consideration of the rotation of the object.

**Figure 13:**
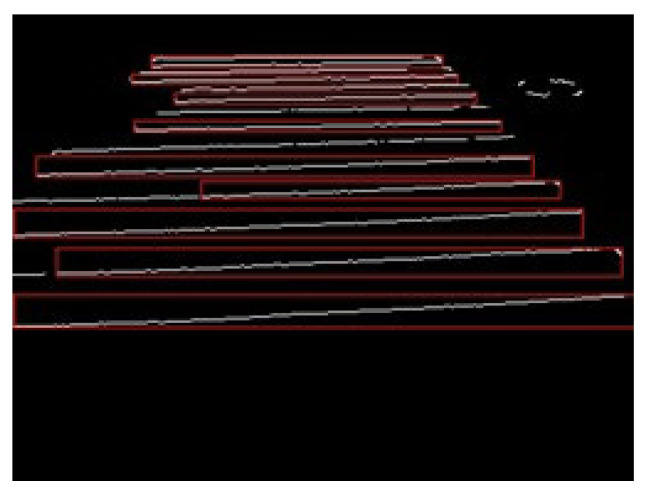
Labeling.

**Figure 14:**
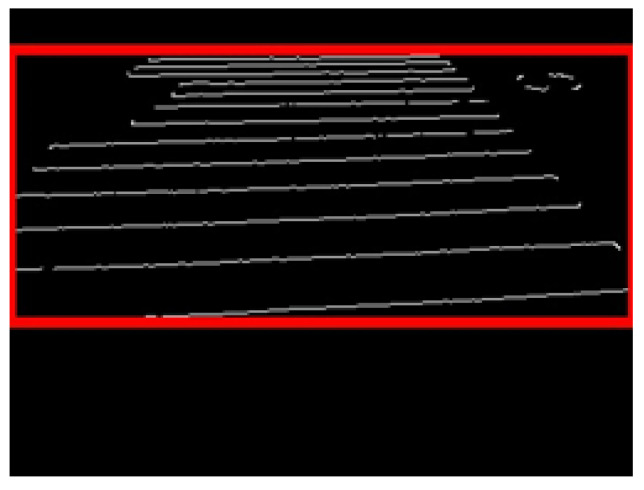
Label combination.

The multiple detected labels were then sorted by size, and only those with large areas were extracted, and the labels were merged to draw the entire crosswalk area. Figure 13 shows the labeling results, and Figure 14 shows the result of merging the labels.

## 5. Experiment

### 5.1. Experimental Environment

We performed experiments using the proposed system and evaluated its performance. The experiments were conducted using 1390 crosswalk images taken during the day in different environments. This means that the images were captured while walking on various pedestrian crossings located on different roads, and under both day and night conditions. We calculated the true positive (TP) and false negative (FN) rates using crosswalk images. In addition, we used 1100 images that did not include pedestrian crossings to confirm the false positive (FP) and true negative (TN) rates.

A similar experiment was conducted using 520 crosswalk images taken at night and 520 images without crosswalks.

The camera was fixed to the chest of the pedestrian during video acquisition. The pedestrian walked at a normal walking speed. We utilized the C++ programming language and the OpenCV libraries for our implementation.

We further verified the real-time performance using the Jetson Nano computer. Table 2 shows information on the Jetson Nano. A 30 s video was processed and the processing time was calculated.

### 5.2. Evaluation

**Figure 15:**
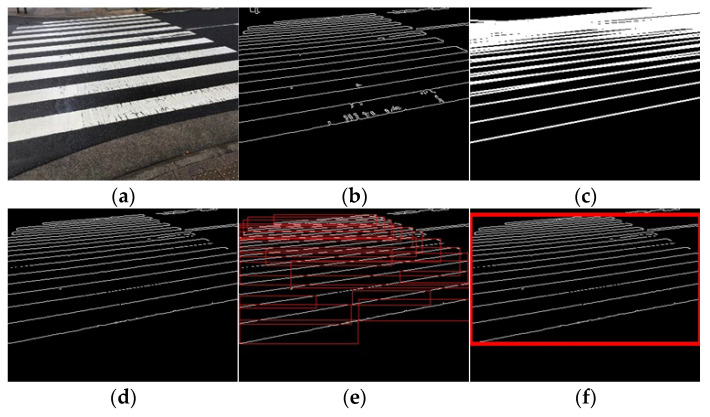
Result 1. (**a**) Input image, (**b**) edge image, (**c**) image of straight line obtained via Hough transform, (**d**) composite image, (**e**) labeling, and (**f**) detection results.

**Figure 16:**
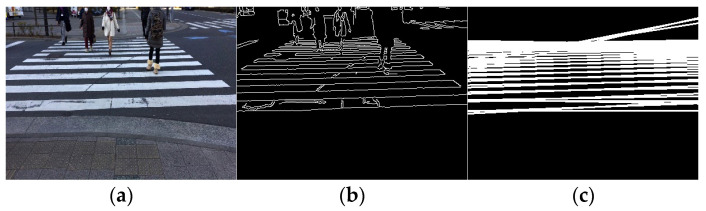

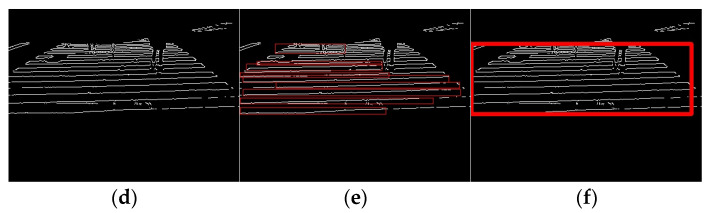
Result 2. (**a**) Input image, (**b**) edge image, (**c**) image of straight line obtained via Hough transform, (**d**) composite image, (**e**) labeling, and (**f**) detection results.

**Figure 17:**
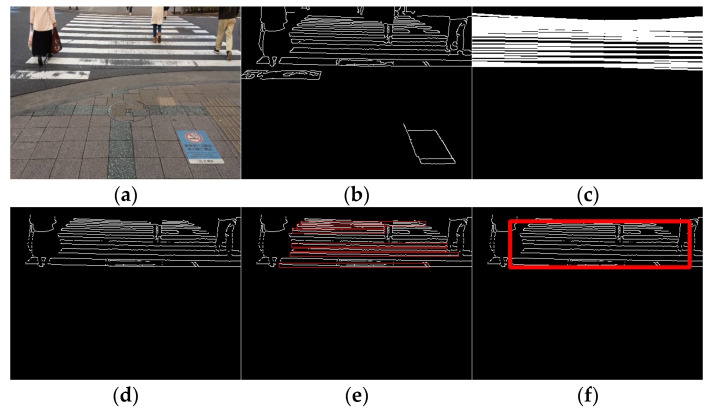
Result 3. (**a**) Input image, (**b**) edge image, (**c**) image of straight line obtained via Hough transform, (**d**) composite image, (**e**) labeling, and (**f**) detection results.

**Figure 18:**
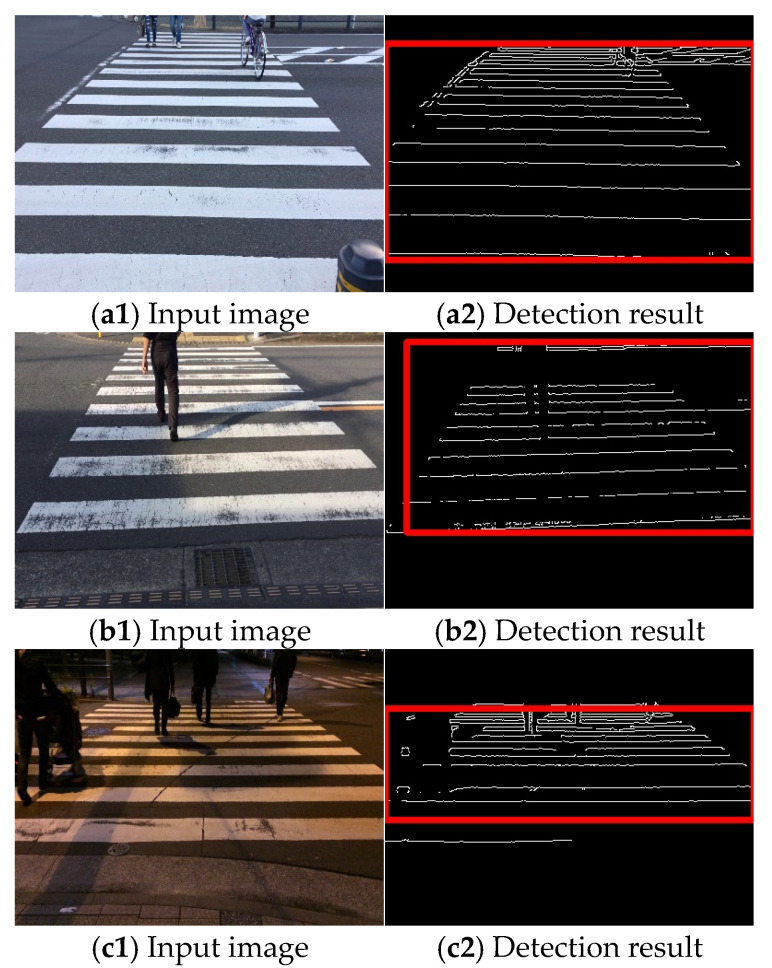
Sensing results 1 ((**left**): input image (**right**): sensing result).

**Figure 19:**
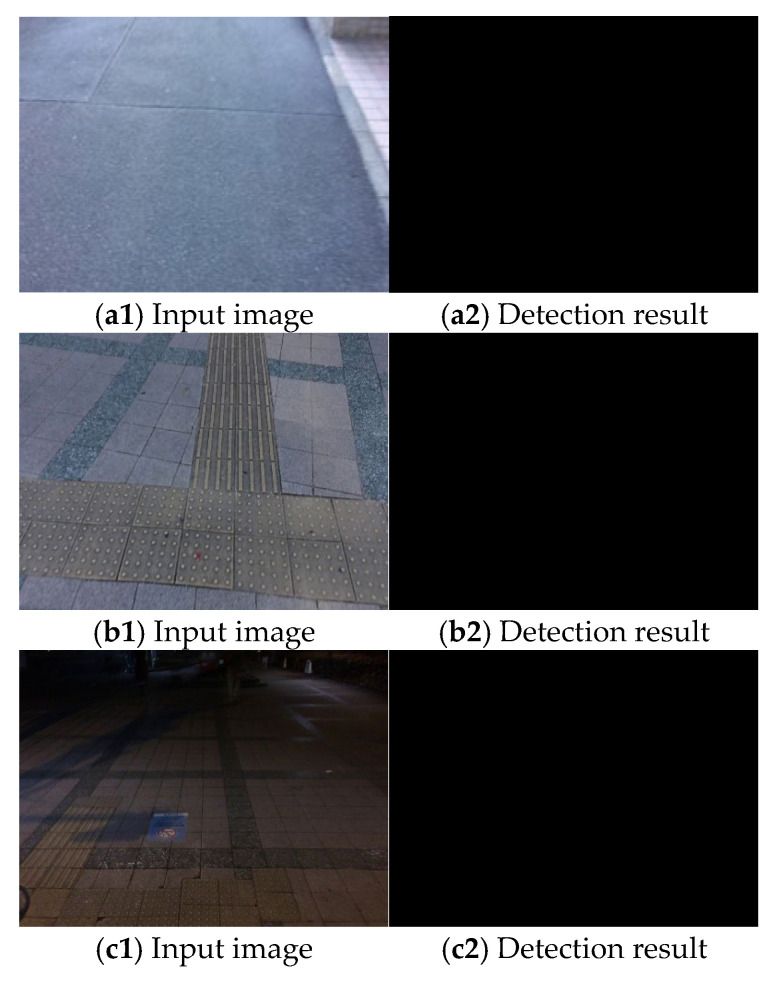
Sensing results 2 ((**left**): input image (**right**): sensing result).

Video acquisition was performed at different times and places, and processing was performed for each input video. The selected results are presented in Figure 15, Figure 16 and Figure 17. Figure 15 shows only the crosswalk. Figure 16 and Figure 17 show pedestrians and the crosswalk, respectively. In each figure, panel (a) shows the input image, panel (b) shows the edge image, panel (c) shows an image in which only the straight lines obtained via Hough transform are extracted, panel (d) shows the composite image, panel (e) shows the labeling, and panel (f) shows the pedestrian crossing sensing results. Figure 18 and Figure 19 show the sensing results for the input and output images.

Thus, it is shown that detection is possible even when the crosswalk is hidden by a person.

If the labeling of parallel lines exceeding 1/100 of the screen size is possible, it can be recognized as a pedestrian crossing. Therefore, it is not the measure of population density, but how it appears from the camera that is important.

Table 3 summarizes the results obtained during the day. Table 4 summarizes the results obtained at night. Table 3 and Table 4 summarize the following evaluation results: TP, TN, FP, and FN. TP and TN correspond to images in which the presence or absence of a pedestrian crossing is correctly recognized. FP and FN correspond to images in which the presence or absence of a pedestrian crossing is not correctly recognized. We evaluated the identification results according to the accuracy, which is summarized in Table 3 and Table 4. The calculation of accuracy was performed following the definition in Equation (12). The accuracy was 98.5% when the proposed algorithm was tested with pedestrian crossing images and images without pedestrian crossing. Hence, the proposed method detected pedestrian crossings in both environments with good accuracy and a comparatively low FP rate. In addition, the accuracy of our results is higher than that of previous studies. For instance, Wu et al. [15] had an average accuracy of 95.3%, and Cao et al. [17] achieved an accuracy of 94.9% (Table 5).

The processing time for a 30 s video at 30 fps was 85.6 s. These results indicate that real-time processing can be performed at approximately 10.5 fps.

Considering the walking speed of a visually impaired person, this performance is considered sufficient.

(12) $$Accuracy=\frac{TP+TN}{TP+TN+FP+FN}$$

### 5.3. Discussion

In this study, a pedestrian crossing was detected based on edge information and the characteristics of the slope of straight lines. Thus, only a minimal amount of ambient light is necessary for detection, and the results are not affected by the light level owing to the weather or time of day. The results are more dependent on the capturing sensitivity of the camera rather than the specific degree of lighting. Modern high-sensitive cameras are capable of capturing objects in images even under low light conditions. Based on the results obtained thus far, if a pedestrian crossing can be captured in images similar to Figure 18(c1), the proposed method has demonstrated successful detection capabilities. We conducted our experiments under non-rainy and non-snowy conditions, both during daytime and nighttime. The proposed method has been found to function successfully under these weather conditions. Therefore, as shown in Figure 15, Figure 16 and Figure 17, the method is considered effective in many cases. Additionally, we succeeded in detecting pedestrian crossings at night.

However, if a significant portion of the pedestrian crossing marking is missing, it will not be detected because no sufficiently straight lines can be detected in the edge image.

Regarding the number of pedestrians on the crossing, we have observed that the appearance of the white regions of the pedestrian crossing changes randomly based on the positions of the pedestrians. Therefore, in our experience, the detection results of the pedestrian crossing depend more on the area of the pedestrian crossing that can be captured in the camera images rather than the exact number of pedestrians. Based on the results obtained thus far, we have observed that if the edges of up to two white layers of the pedestrian crossing can be detected, even when pedestrians are present on those layers, this method can successfully detect a pedestrian crossing.

On the other hand, the proposed method primarily detects pedestrian crossings based on the parallel lines associated with the edges of the parallel white layers of a pedestrian crosswalk. This indicates that as long as the camera can capture the pedestrian crossing from any direction in the images, the proposed method will work effectively. The appearance of the parallel layers on the images is independent of the capturing direction.

## 6. Conclusions

In this study, we developed a pedestrian crossing detection method to assist visually impaired pedestrians to walk safely. Our proposed detection method detects edges in an image, processes the edge information in Hough space, and analyzes the variance of the edge inclination in Hough space. Our proposed method has unique characteristics that make it effective in handling challenging scenarios where traditional methods struggle. It excels at detecting crosswalks in low-light conditions, even when visibility is limited or obstructed. This method achieves high real-time performance by minimizing computational costs, ensuring the efficient and timely detection of crosswalks in real-world environments. It demonstrates an impressive accuracy rate of 98.47%. The algorithm can be executed at almost real-time speeds (approximately 10.5 fps) using a Jetson Nano small-type computer, highlighting its potential as a wearable device. Conducting a wide range of subjective experiments with visually impaired individuals using the proposed method and dedicated hardware will be a key focus of our future work.

## Figures and Tables

**Table 1:** Camera Specifications.

Item	Value
Frame width	1920 pixels
Frame height	1080 pixels
Frame	30 fps
Aperture size	F1.8
Focal length	28 mm
Sensor size	1/3
Pixel size	1.22 μm

**Table 2:** Jetson Nano specifications.

Item	Value
CPU	ARMA57@1.43 GHz 4 core
GPU	128-core Maxwell
Memory	4 GB 64-bit LPDDR4 25.6 GB/s
Storage	microSD 64 GB
USB	4× USB3.0
Power Input	5 V, MAX 4 A
Mechanical	69 mm × 45 mm

**Table 3:** Evaluation result of the pedestrian crossing detection method (daytime).

		Actuality	
		Present	Absent
**Predicted**	**Crosswalk**	1385 (TP)	33 (FN)
	**No Crosswalk**	5 (FP)	1067 (TN)
**Accuracy**	**(TP + TN)/(P + N)**	98.5%	

**Table 4:** Evaluation result of the pedestrian crossing detection method (night).

		Actuality	
		Present	Absent
**Predicted**	**Crosswalk**	468 (TP)	0 (FN)
	**No Crosswalk**	52 (FP)	520 (TN)
**Accuracy**	**(TP + TN)/(P + N)**	95.0%	

**Table 5:** Comparison of accuracy.

Methods	Accuracy
Wu et al. [15]	95.3%
Cao et al. [17]	94.9%
This method	98.5%

## Data Availability

Not applicable.

## References

[1] Japanese Service Dog Resource Center. https://www.jsdrc.jp/hojoken/modoken.

[2] Kansai Guide Dogs for the Blind Association. https://kansai-guidedog.jp/knowledge/activity/index.html.

[3] Chen L., Zhang Y., Miao S., Zhu S., Hu R., Peng L., Lv M. (2022). SALIENCE: An Unsupervised User Adaptation Model for Multiple Wearable Sensors Based Human Activity Recognition. IEEE Trans. Mob. Comput..

[4] Matsumura H., Premachandra C. (2022). Deep-Learning-Based Stair Detection Using 3D Point Cloud Data for Preventing Walking Accidents of the Visually Impaired. IEEE Access.

[5] Fan Y., Sun Z., Zhao G. (2020). A Coarse-to-Fine Framework for Multiple Pedestrian Crossing Detection. Sensors.

[6] Choi J., Miyoshi M., Ishikawa S., Morie T. (2010). Detecting Pedestrians on a Zebra Crossing from Car Video Images. Biomed. Fuzzy Syst. Assoc..

[7] Hernández D.C., Filonenko A., Seo D., Jo K. Crosswalk detection based on laser scanning from moving vehicle. Proceedings of the 2015 IEEE 13th International Conference on Industrial Informatics (INDIN).

[8] Choi J., Ahn B.T., Kweon I.S. Crosswalk and traffic light detection via integral framework. Proceedings of the 19th Korea-Japan Joint Workshop on Frontiers of Computer Vision.

[9] Malbog M.A. MASK R-CNN for Pedestrian Crosswalk Detection and Instance Segmentation. Proceedings of the 2019 IEEE 6th International Conference on Engineering Technologies and Applied Sciences (ICETAS).

[10] Suzuki S., Raksincharoensak P., Shimizu I., Nagai M., Adomat R. Sensor fusion-based pedestrian collision warning system with crosswalk detection. Proceedings of the 2010 IEEE Intelligent Vehicles Symposium.

[11] Haselhoff A., Kummert A. On visual crosswalk detection for driver assistance systems. Proceedings of the 2010 IEEE Intelligent Vehicles Symposium.

[12] Zhai Y., Cui G., Gu Q., Kong L. Crosswalk Detection Based on MSER and ERANSAC. Proceedings of the 2015 IEEE 18th International Conference on Intelligent Transportation Systems.

[13] Llorca D.F., Parra I., Quintero R., Fernández C., Izquierdo R., Sotelo M.A. Stereo-based pedestrian detection in crosswalks for pedestrian behavioural modelling assessment. Proceedings of the 2014 11th International Conference on Informatics in Control, Automation and Robotics (ICINCO).

[14] Cheng R., Wang K., Yang K., Long N., Hu W., Chen H., Bai J., Liu D. (2017). Crosswalk navigation for people with visual impairments on a wearable device. J. Electron. Imaging.

[15] Wu X., Hu R., Bao Y. (2019). Block-Based Hough Transform for Recognition of Zebra Crossing in Natural Scene Images. IEEE Access.

[16] Radványi M., Varga B., Karacs K. Advanced crosswalk detection for the Bionic Eyeglass. Proceedings of the 2010 12th International Workshop on Cellular Nanoscale Networks and their Applications (CNNA 2010).

[17] Cao Y., Chen L., Jia S. An Image Based Detection of Pedestrian Crossing. Proceedings of the 2009 2nd International Congress on Image and Signal Processing.

[18] Akbari Y., Hassen H., Subramanian N., Kunhoth J., Al-Maadeed S., Alhajyaseen W. A vision-based zebra crossing detection method for people with visual impairments. Proceedings of the 2020 IEEE International Conference on Informatics, IoT and Enabling Technologies (ICIoT).

[19] Mascetti S., Ahmetovic D., Gerino A., Bernareggi C. (2016). ZebraRecognizer: Pedestrian crossing recognition for people with visual impairment or blindness. Pattern Recognit..

[20] Silva E.T., Sampaio F., Silva L.C., Medeiros D.S., Correia G.P. (2020). A method for embedding a computer vision application into a wearable device. Microprocess. Microsyst..

[21] Ivanchenko V., Coughlan J., Shen H. Detecting and locating crosswalks using a camera phone. Proceedings of the 2008 IEEE Computer Society Conference on Computer Vision and Pattern Recognition Workshops.

[22] Romić K., Galić I., Leventić H., Habijan M. SVM based column-level approach for crosswalk detection in low-resolution images. Proceedings of the 2020 International Symposium ELMAR.

[23] Haider M.M., Hoque M.R., Khaliluzzaman M., Hassan M.M. Zebra Crosswalk Region Detection and Localization Based on Deep Convolutional Neural Network. Proceedings of the 2019 IEEE International Conference on Robotics, Automation, Artificial-Intelligence and Internet-of-Things (RAAICON).

[24] Shioyama T., Wu H., Nishibe Y., Nakamura N., Kitawaki S. Image analysis of crosswalk. Proceedings of the 11th International Conference on Image Analysis and Processing.

[25] Cao Z., Xu X., Hu B., Zhou M. (2020). Rapid Detection of Blind Roads and Crosswalks by Using a Lightweight Semantic Segmentation Network. IEEE Trans. Intell. Transp. Syst..

[26] Son H., Krishnagiri D., Jeganathan V.S., Weiland J. Crosswalk Guidance System for the Blind. Proceedings of the 2020 42nd Annual International Conference of the IEEE Engineering in Medicine & Biology Society (EMBC).

[27] Wang S., Tian Y. Detecting stairs and pedestrian crosswalks for the blind by RGBD camera. Proceedings of the 2012 IEEE International Conference on Bioinformatics and Biomedicine Workshops.

[28] Hough P. (1962). Method and Means for Recognizing Complex Patterns.

[29] Canny J. (1986). A Computational Approach to Edge Detection. IEEE Trans. Pattern Anal. Mach. Intell..

[30] Hao G., Min L., Feng H. Improved Self-Adaptive Edge Detection Method Based on Canny. Proceedings of the 2013 5th International Conference on Intelligent Human-Machine Systems and Cybernetics.

[31] Lu H., Yan J. Window frame obstacle edge detection based on improved Canny operator. Proceedings of the 2019 3rd International Conference on Electronic Information Technology and Computer Engineering (EITCE).

[32] Akinlar C., Chome E. CannySR: Using smart routing of edge drawing to convert Canny binary edge maps to edge segments. Proceedings of the 2015 International Symposium on Innovations in Intelligent SysTems and Applications (INISTA).

[33] Shanmugavadivu P., Kumar A. Modified Eight-Directional Canny for Robust Edge Detection. Proceedings of the 2014 International Conference on Contemporary Computing and Informatics (IC3I).

[34] Raghavendra V., Shrinivasan L. Time Efficient Design and FPGA Implementation of Distributed Canny Edge Detector Algorithm. Proceedings of the 2018 3rd IEEE International Conference on Recent Trends in Electronics, Information & Communication Technology (RTEICT).

[35] Sobel I. (1970). An Isotropic 3 × 3 Image Gradient Operator. The Stanford Artificial Intelligence Laboratory Memo AIM-160.

[36] Ito Y., Premachandra C., Sumathipala S., Premachandra H.W.H., Sudantha B.S. (2021). Tactile Paving Detection by Dynamic Thresholding Based on HSV Space Analysis for Developing a Walking Support System. IEEE Access.

[37] Premachandra H.W.H., Premachandra C., Parape C.D., Kawanaka H. (2017). Speed-up Ellipse Enclosing Character Detection Approach for Large-size Document Images by Parallel Scanning and Hough Transform. Int. J. Mach. Learn. Cybern..

[38] Premachandra C., Gohara R., Kato K. Fast Lane Boundary Recognition by a Parallel Image Processor. Proceedings of the 2016 IEEE International Conference on Systems, Man, and Cybernetics.

[39] Premachandra C., Ueda D., Kato K. (2019). Speed-up Automatic Quadcopter Position Detection by Sensing Propeller Rotation. IEEE Sens. J..

[40] Guo S., Kong Y., Tang Q., Zhang F. Probabilistic Hough transform for line detection utilizing surround suppression. Proceedings of the 2008 International Conference on Machine Learning and Cybernetics.

